# StMPK7 phosphorylates and stabilizes a potato RNA-binding protein StUBA2a/b to enhance plant defence responses

**DOI:** 10.1093/hr/uhac177

**Published:** 2022-08-24

**Authors:** Tingting Li, Haizhu Zhang, Liwen Xu, Xiaokang Chen, Jiashu Feng, Weijun Wu, Yu Du

**Affiliations:** College of Horticulture, Northwest A&F University and State Key Laboratory of Crop Stress Biology for Arid Areas, Yangling 712100, China; College of Horticulture, Northwest A&F University and State Key Laboratory of Crop Stress Biology for Arid Areas, Yangling 712100, China; College of Horticulture, Northwest A&F University and State Key Laboratory of Crop Stress Biology for Arid Areas, Yangling 712100, China; College of Horticulture, Northwest A&F University and State Key Laboratory of Crop Stress Biology for Arid Areas, Yangling 712100, China; College of Horticulture, Northwest A&F University and State Key Laboratory of Crop Stress Biology for Arid Areas, Yangling 712100, China; College of Horticulture, Northwest A&F University and State Key Laboratory of Crop Stress Biology for Arid Areas, Yangling 712100, China; College of Horticulture, Northwest A&F University and State Key Laboratory of Crop Stress Biology for Arid Areas, Yangling 712100, China

## Abstract

Mitogen-activated protein kinase (MAPK) cascades play pivotal roles in regulating plant immunity. MAPKs usually transduce signals and regulate plant immunity by phosphorylating the downstream defence-related components. Our previous study indicates that StMPK7 positively regulates plant defence to *Phytophthora* pathogens via SA signalling pathway. However, the downstream component of StMPK7 remains unknown. In this study, we employed *GFP-StMPK7* transgenic potato and performed immunoprecipitation-mass spectrometry (IP-MS) to identify the downstream component of StMPK7. We found that an RNA binding protein StUBA2a/b interacted with StMPK7, as revealed by luciferase complementation imaging (LCI) and coimmunoprecipitation (co-IP) assays. Transient expression of *StUBA2a/b* in *Nicociana benthamiana* enhanced plant resistance to *Phytophthora* pathogens, while silencing of *UBA2a/b* decreased the resistance, suggesting a positive regulator role of UBA2a/b in plant immunity. Similar to StMPK7, StUBA2a/b was also involved in SA signalling pathway and induced SGT1-dependent cell death as constitutively activated (CA)-StMPK7 did. Immune blotting indicated that StMPK7 phosphorylates StUBA2a/b at thr248 and thr408 (T248/408) sites and stabilizes StUBA2a/b. Silencing of *MPK7* in *N*. *benthamiana* suppressed StUBA2a/b-induced cell death, while co-expression with *StMPK7* enhanced the cell death. Besides, StUBA2a/b^T248/408A^ mutant showed decreased ability to trigger cell death and elevate the expression of *PR* genes, indicating the phosphorylation by StMPK7 enhances the functions of StUBA2a/b. Moreover, CA-StMPK7-induced cell death was largely suppressed by silencing of *NbUBA2a/b*, genetically implying UBA2a/b acts as the downstream component of StMPK7. Collectively, our results reveal that StMPK7 phosphorylates and stabilizes its downstream substrate StUBA2a/b to enhance plant immunity via the SA signalling pathway.

## Introduction

Potato (*Solanum tuberosum*) is recommended as a food security crop by the Food and Agriculture Organization (FAO, 2014) and is one of the most economically important crops in terms of global consumption. Potato late blight caused by *Phytophthora infestans* is considered the most devastating disease in potato and leads to billions of dollars of economic losses every year [1, 2]. The rapid variation and high genetic diversity of *Phytophthora* pathogens lead to serious problems in controlling the diseases, such as strong drug resistance and easy loss of crop resistance [3]. Uncovering the mechanism of plant defence to these pathogens and utility of host defence to improve potato breeding is critical for the effective and eco-friendly control of the diseases caused by these pathogens.

In the threats of various potential pathogens, plants have evolved an effective defence system to sense and defend against invading pathogens. During infection, pathogen/microbe-associated molecular patterns (PAMPs/MAMPs) or apoplastic pathogen effectors are recognized by cell-surface immune receptors and lead to pattern-triggered immunity (PTI), whereas pathogen intracellular effectors are perceived by plant intracellular nucleotide-binding, leucine-rich repeat receptors (NLRs) to induce effector-triggered immunity (ETI) [4]. Both PTI and ETI induce common downstream signalling events, including altered calcium flux, production of reactive oxygen species (ROS), activation of mitogen-activated protein kinases (MAPKs) cascade, transcriptional reprogramming and production of defence hormones [4–6].

Plant MAPK cascade is a conserved signalling pathway and plays a central role in defence [7, 8]. MAPKs are activated by their upstream MAPK kinases (MAPKKs or MEKs), which in turn are phosphorylated by MAPKK kinases or MEK kinases (MAPKKKs or MEKKs) [9]. By phosphorylating different downstream substrates, MAPKs can regulate various plant defence responses including the signalling of defence hormones, defence-related gene expression, ROS generation, and hypersensitive response (HR) cell death [7, 10]. AtMPK3, AtMPK6, and AtMPK4 are the best-characterized MAPKs in Arabidopsis and have shown to be important components in plant immunity [8]. AtMPK3 and AtMPK6 function redundantly in the same MAPK cascade and enhance plant defence to a wide range of pathogens via phosphorylation of different substrates, including a subset of ethylene biosynthesis-related ACC synthases [11, 12], the ethylene response factor ERF104 [13], ERF6 [14], WRKY33 that is essential for the induction of camalexin biosynthesis [15, 16], the calmodulin-binding transcription activator CAMTA3 [17] and the major ETI regulator SGT1 [18]. AtMPK4 plays complex roles in plant immunity and also targets multiple downstream substrates. For example, a positive regulator of plant defence MKS1 was identified as the substrates of AtMPK4 [19, 20]. Calmodulin-binding receptor-like cytoplasmic kinase CRCK3 was identified as the substrate of MPK4 and its phosphorylation was monitored by an NLR protein SUMM2 [21]. A plant-specific trihelix transcription factor ASR3 was also phosphorylated by MPK4 and hence resulting in enhanced DNA binding activity [22]. These previous studies show that the MAPK signalling pathways regulate plant defence by phosphorylation of transcription factors, enzymes, or some plant immune regulators. A few studies also identified RNA-binding proteins (RBPs) as the targets of MAPKs to regulate PTI responses. For example, the *Arabidopsis thaliana* tandem zinc finger protein 9 (TZF9) was phosphorylated by MPK3 and MPK6 to positively regulate plant defence to *Pseudomonas syringae* pv. tomato DC3000 [23, 24].

RBPs are characterized by the containing of RNA-binding motifs and participate in all steps of RNA processing through binding with their RNA targets [25, 26]. The Arabidopsis heterogeneous nuclear ribonucleoprotein (hnRNP) UBP1-associated protein 2 (UBA2) is homologous to *Vicia faba* AAPK interacting protein 1 (VfAKIP1), which is phosphorylated by an ABA-activated serine–threonine-protein kinase (AAPK) [27–29]. StUBA2a/b in potato is the ortholog of AtUBA2a, AtUBA2b, and VfAKIP1. It induces hypersensitive-like cell death in *Nicotiana tabacum* leaves and early leaf senescence in Arabidopsis via increasing the defence- and senescence-associated gene expressions [30]. However, the role of StUBA2a/b itself in plant defence to pathogens has not been investigated.

The roles of MAPK cascades in plant defence are well studied in model plants. However, only a few studies in potato investigated the roles of MAPK cascade proteins in plant immunity. For example, potato mitogen-activated protein kinase kinases StMAP3Kβ2 and StMAP3Kε function in parallel in the same signal transduction pathway that positively regulates potato immunity [31], while the MEK kinase StVIK was reported as a negative regulator of potato immunity [32]. Our previous studies showed that *P. infestans* RXLR effector targets and stabilizes StMKK1 to facilitate pathogen colonization [33]. Further investigation reveals that StMKK1 negatively regulates potato immunity to biotrophic and hemi-biotrophic pathogens by repressing PTI and SA-related signaling pathways [34]. The StMKK1 downstream signalling target StMPK7 was found to positively regulate plant defence to *Phytophthora* pathogens via the SA signalling pathway [35]. However, how StMPK7 participates in plant SA-related immunity remains unknown. In this study, we performed experiments to search for the downstream signalling target proteins of StMPK7 in order to reveal the underlying mechanisms of StMPK7-activated potato immunity. We treated the potato StMPK7-GFP-overexpression lines with flg22, and at 15 min after flg22 treatment the leaves were harvested to perform the immunoprecipitation-mass spectrometry (IP-MS). The RNA binding protein StUBA2a/b was identified as the potential interacting protein of StMPK7. Further investigations showed that StMPK7 interacts with and phosphorylates StUBA2a/b, and UBA2a/b positively regulates plant defence via the SA signalling pathway. Thus, we conclude that StMPK7 phosphorylates and stabilizes StUBA2a/b, which positively regulates the SA signalling pathway, to enhance plant immunity.

## Results

### StMPK7 interacts with an RNA binding protein StUBA2a/b

**Figure 1:**
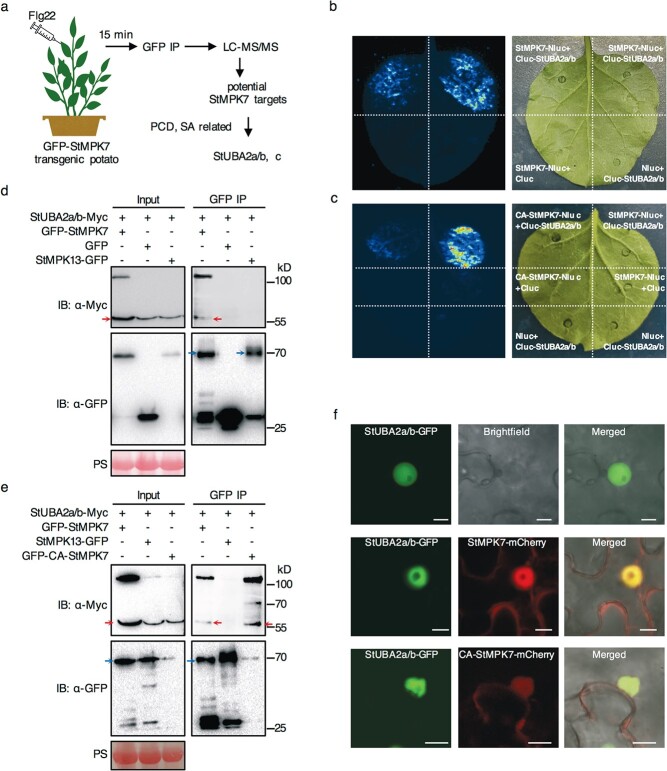
StMPK7 and CA-StMPK7 interact with an RNA binding protein StUBA2a/b. **a** Workflow of the method to identify the potential StMPK7 targets. The protein was extracted from middle leaves of GFP-StMPK7 transgenic potato treated with 20 μM flg22 for 15 min and subsequently subjected to GFP Immunoprecipitation (IP) combined with liquid chromatography with tandem-mass spectrometry (LC–MS/MS). Firefly luciferase complementation imaging (LCI) assays showed that StUBA2a/b interacts with both StMPK7 (**b**) and CA-StMPK7 (**c**). Nluc with Cluc-StUBA2a/b, CA-StMPK7-Nluc with Cluc, and StMPK7-Nluc with Cluc were used as the negative controls. The left panel was the images taken by CCD-camera while the right panel was photos taken under normal light. Coimmunoprecipitation (co-IP) assays showed the interaction between StUBA2a/b-Myc with both GFP-StMPK7 (**d**) and GFP-CA-StMPK7 (**e**). Total proteins were extracted with *N. benthamiana* leaves expressing the corresponding constructs as indicated by ‘+’ at 2 days post agro-infiltration (dpi). IP was performed via GFP-Trap beads. Red arrows indicate the intact protein of StUBA2a/b-Myc. Blue arrows mark the intact protein of GFP tagged StMPK7, StMPK13, and CA-StMPK7. Free GFP and StMPK13-GFP were used as the negative controls. ‘+’ and ‘-‘ indicates the presence and absence of the construct in the samples. The protein loading is shown by Ponceau staining (PS). **f** Subcellular localization of StUBA2a/b-GFP and co-localization of StUBA2a/b-GFP and StMPK7-mCherry or CA-StMPK7-mCherry in *N. benthamiana*. The fluorescence imaging was visualized at 2 dpi. Scale bars, 10 μm.

Because StMPK7 was previously found to activate SA-related immunity, and SA is required for plant immunity to biotrophic and hemibiotrophic pathogens, we thus hypothesize that StMPK7-transgenic lines may enhance potato immunity to other pathogens besides *P. infestans*. To prove this, we inoculated the StMPK7-overexpression transgenic potato lines with bacterial wilt pathogen *Ralstonia solanacearum*. The wilting symptoms and bacterial growth quantifications indicate that StMPK7 enhances potato resistance to this bacterial pathogen (Fig. S1, see online supplementary material). To understand the mechanism of how StMPK7 activates SA-related immunity, we used an IP-MS approach to identify the downstream substrate of StMPK7. The potato GFP-StMPK7 transgenic lines were treated with flg22 and at 15 min after flg22 treatment, the samples were harvested to identify the GFP-StMPK7-interacting proteins (Fig. 1a). A total of 297 potential interactors of StMPK7 were obtained. Among them, two of the five RNA-binding proteins (Table S1, see online supplementary material), StUBA2a/b and StUBA2c [30], are reported to trigger SA-related plant cell death (CD) in tobacco leaves, which is also observed in CA-StMPK7 expressing *N*. *benthamiana* leaves [35]. We thus selected StUBA2a/b as a potential target of StMPK7 (Fig. 1a and Table S1, see online supplementary material) for further study. To confirm the interaction between StMPK7 and StUBA2a/b, we performed firefly luciferase complementation imaging (LCI) assay in *N*. *benthamiana* leaves. As shown in Fig. 1b, luciferase activity was detected in the area co-expressing StMPK7-Nluc and Cluc-StUBA2a/b under blue light, but not in that co-expressing the negative controls (StMPK7-Nluc with Cluc; Cluc-StUBA2a/b with Nluc) (Fig. 1b and Fig. S2a, see online supplementary material). We also examined the interaction between CA-StMPK7 and StUBA2a/b by LCI. Co-expression of CA-StMPK7-Nluc and Cluc-StUBA2a/b leads to an obvious luciferase activity, although weaker than StMPK7-Nluc and Cluc-StUBA2a/b coexpression (Fig. 1c and Fig. S2b, see online supplementary material). The expression of StMPK7-Nluc, CA-StMPK7-Nluc, and Cluc-StUBA2a/b proteins fused with Myc tag was confirmed by immunoblotting with anti-Myc antibody (Fig. S2c, see online supplementary material).

Further coimmunoprecipitation (co-IP) assay indicated that StUBA2a/b-Myc could coimmunoprecipitate with both GFP-StMPK7 (Fig. 1d) and GFP-CA-StMPK7 (Fig. 1e), but not with the negative control GFP or StMPK13-GFP [36]. Reverse co-IP also confirmed the interaction between Myc-StMPK7 and StUBA2a/b-GFP (Fig. S2d and e, see online supplementary material). To examine the subcellular co-localization of StUBA2a/b with StMPK7 or CA-StMPK7, we co-expressed StUBA2a/b-GFP with StMPK7-mCherry or CA-StMPK7-mCherry in *N. benthamiana*, respectively. The confocal microscopy images showed that StUBA2a/b-GFP localized specifically in the nucleus (Fig. 1f, upper panel), and it can co-localize with both StMPK7-mCherry (Fig. 1f, middle panel) and CA-StMPK7-mCherry (Fig. 1f, lower panel) in the nucleus. Taken together, these results indicate both StMPK7 and CA-StMPK7 interact with StUBA2a/b.

### UBA2a/b positively regulates plant resistance to *Phytophthora* pathogens

**Figure 2:**
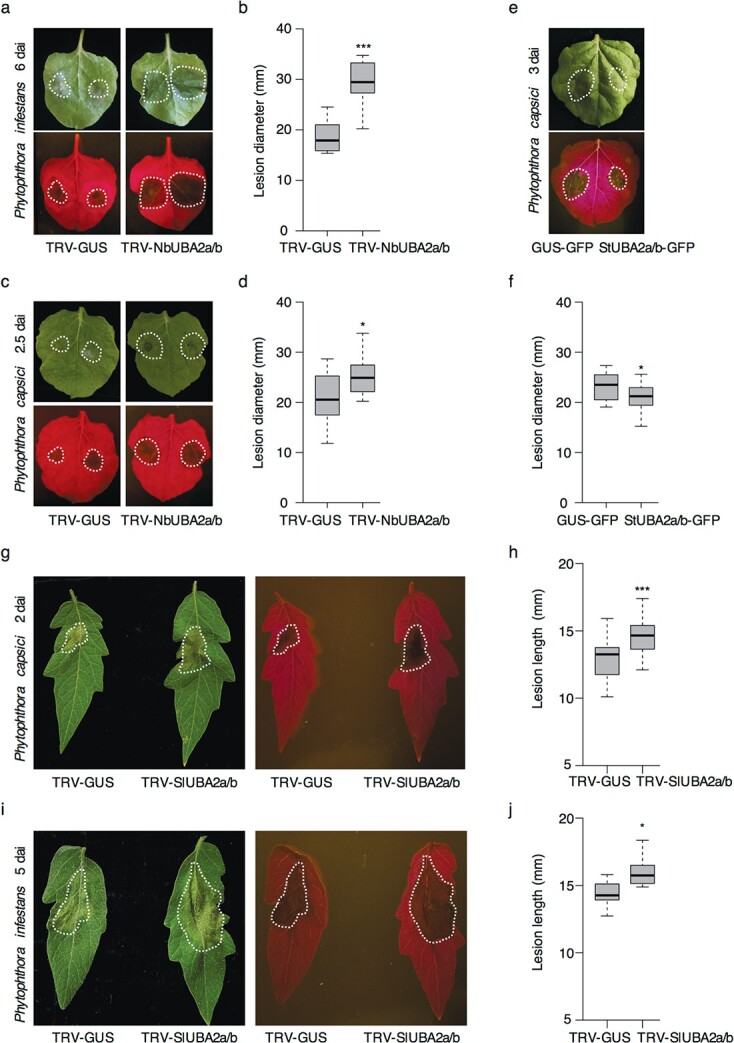
UBA2a/b positively regulates plant resistance to *Phytophthora* pathogens. **a**–**d** Silencing of *NbUBA2a/b* decreased plant resistance to *P. infestans* (**a**, **b**) and *P. capsici* (**c**, **d**). The lesions developed in TRV-*NbUBA2a/b* leaves at 6 days after infection (dai) by *P. infestans* (**a**) or at 2.5 dai by *P. capsici* (**c**) were larger than that in the control leaves of TRV-*GUS*, as indicated by the representative photographs taken under normal (upper panel) or blue light (lower panel). Lesion diameter for *P. infestans* (**b**) and *P. capsici* (**d**) infection were shown by the boxplots. Four to six leaves (two infection sites in each leave) from independent TRV-*GUS* or TRV-*NbUBA2a/b* plants were used for inoculation. Statistical analyses were performed with one-sided *t*-tests (^*^, *P* < 0.05; ^***^, *P* < 0.001; *n* ≥ 8). **e**, **f** Transient expression of StUBA2a/b in *N. benthamiana* enhanced plant resistance to *P. capsici*. StUBA2a/b-GFP or GUS-GFP was agro-infiltrated into the right or left side of *N. benthamiana* leaves. *P. capsici* was inoculated onto the leaves 6 hours post-agro-inoculation and photographs were taken at 3 dai under normal (upper panel) or blue light (lower panel). Lesion diameters from 8 infection sites (one infection site in each leave) were shown by the boxplot in (**f**). Paired *t*-test was used in the statistical analysis (^*^, *P* < 0.05; *n* = 8). Images of the representative leaves from TRV-*SlUBA2a/b* and TRV-*GUS* plants infected by *P. capsici* at 2 dai (**g**) and *P. infestans* at 5 dai (**i**). The photos were taken under normal light (left panel) and blue light (right panel). Lesion length analyses for *P. capsici* (**h**) and *P. infestans* (**i**) infection showing larger lesions developed in the TRV-*SlUBA2a/b* leaves than in the control TRV-GUS. Boxplot shows the data from at least eight independent TRV-*SlUBA2a/b* or TRV-*GUS* plants (2–4 leaves were obtained from each plant). Statistical analysis was carried out with one-sided *t*-tests (^***^,*P* < 0.001; *n* ≥ 16). Lesion areas are marked with white dotted lines. The experiments were repeated more than two times with similar results.

To investigate the role of UBA2a/b in plant resistance to *Phytophthora* pathogens, we silenced the two copies of *NbUBA2a/b* (Niben101Scf05519g01004.1 and Niben101Scf01001g07022.1) simultaneously in *N*. *benthamiana* using a conserved target region by virus-induced gene silencing (VIGS). Real-time quantitative reverse transcription-PCR (qRT-PCR) data indicated that *NbUBA2a/b* was silenced efficiently in plants expressing TRV-*NbUBA2a/b* as compared to the TRV-*GUS* control (Fig. S3a, see online supplementary material), and the *NbUBA2a/b*-silenced plants did not show any obvious developmental phenotype change (Fig. S3b, see online supplementary material). We inoculated the detached leaves from TRV-*NbUBA2a/b* and TRV-*GUS* plants with *P. infestans* or *Phytophthora capsici*. The lesions developed in the TRV-*NbUBA2a/b* leaves were significantly larger than that in the TRV-*GUS* control (Fig. 2a–d), suggesting the positive regulator role of UBA2a/b in plant defence to *Phytophthora* pathogens. To confirm this, we transiently expressed *StUBA2a/b* in *N*. *benthamiana* (Fig. S2c, see online supplementary material) and inoculated the detached leaves with *P. capsici* at 6 hours post-agro-infiltration. The lesion diameters measured at 3 days after inoculation (dai) in leaves expressing StUBA2a/b-GFP were smaller than those expressing GUS-GFP (Fig. 2e and f), further suggesting the enhancement of plant resistance by StUBA2a/b.

To investigate whether the role of UBA2a/b in plant defence is conserved in other plant, we silenced *SlUBA2a/b* in tomato by VIGS (Fig. S5, see online supplementary material) and performed infection assays. The leaves of *SlUBA2a/b*-silenced tomato showed increased *P. capsici* (Fig. 2g and h) or *P. infestans* (Fig. 2i and j) colonisation compared to the control TRV-GUS leaves, as measured by the lesion length at 2 dai or 5 dai, respectively. Besides, we obtained the Arabidopsis T-DNA mutant *Atuba2a* (SALK_053281) from AraShare (Arabidopsis mutants sharing centre) to investigate the role of AtUBA2a. qRT-PCR was performed to confirm the knockout of *AtUBA2a* (AT3G56860) (Fig. S4a, see online supplementary material), which is the StUBA2a/b ortholog in Arabidopsis. The morphology of *Atuba2a* is similar to the wild type Col-0 (Fig. S4b, see online supplementary material). Detached leaves inoculation assay showed that *Atuba2a* was more susceptible to *P. capsici* than Col-0, suggesting a positive regulator role of *AtUBA2a* in plant defence (Fig. S4c and d, see online supplementary material).

### UBA2a/b is involved in the SA signalling pathway

**Figure 3:**
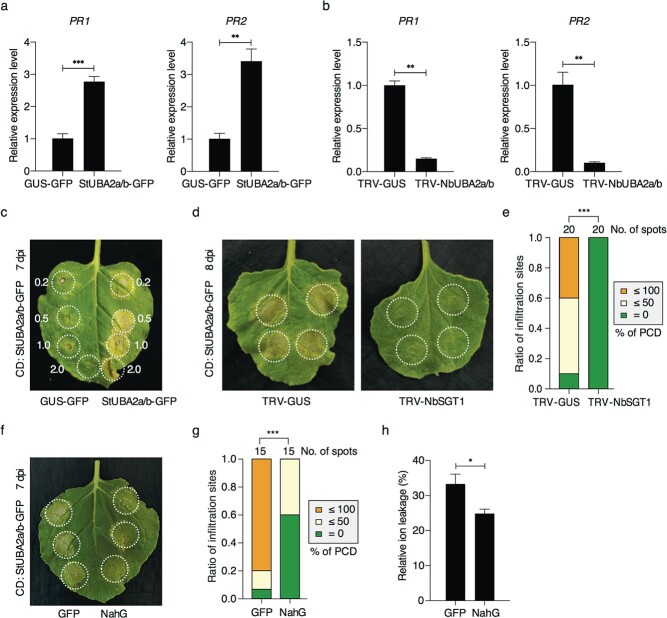
UBA2a/b positively regulates SA signalling pathway. **a** Transient expression of StUBA2a/b in *N. benthamiana* promotes *PR* gene expressions. Total RNAs were extracted from *N. benthamiana* leaves expressing StUBA2a/b-GFP or GUS-GFP for 2 days, and each sample was prepared with four leaves. **b** Silencing of *NbUBA2a/b* in *N. benthamiana* decreased the expression level of *PR* genes. *N. benthamiana* was agro-infiltrated with TRV-*NbUBA2a/b* or TRV-*GUS*, respectively. The middle leaves from four independent TRV-*NbUBA2a/b* or TRV-*GUS* plants at 3 weeks after agro-infiltration were used for RNA extraction. The expression levels of *NbPR1* and *NbPR2* were quantified by qRT-PCR. *NbACTIN* was used as the reference gene. Statistical analyses were performed with one-sided *t*-tests (^**^, *P* < 0.01; ^***^, *P* < 0.001). Error bars indicate the standard deviations. **c** The cell death induced by StUBA2a/b-GFP in *N. benthamiana* leaves. The final agrobacterial suspension culture densities of 0.2, 0.5, 1.0, and 2.0 (OD_600_) were used in the infiltration. **d** Representative photographs of StUBA2a/b-induced cell death (CD) in *NbSGT1*-silenced and the control TRV-GUS *N*. *benthamiana*. *StUBA2a/b*-*GFP* (OD_600_ = 1.0) was infiltrated into the middle leaves of TRV-*NbSGT1* or TRV-*GUS* plants. Photographs were recorded at 8 dpi. **e** Statistical analysis of the StUBA2a/b-induced CD levels in TRV-*NbSGT1* and TRV-*GUS* plants. The CD levels were defined by the percentage of necrotic area in the infiltration area and recorded at 8 dpi. **f**–**h** StUBA2a/b induced CD is suppressed by the salicylate hydroxylase NahG. StUBA2a/b-GFP was co-expressed with NahG or GFP control. The representative photographs were taken at 7 dpi (**f**). Both CD level analysis (**g**) and the relative ion leakage assay (**h**) showed the significant suppression of StUBA2a/b-induced CD when co-expressing with NahG. The white circles mark agro-infiltration area. The total number of infiltration sites was shown above the columns in (**e**) and (**g**). One-sided Wilcoxon rank-sum tests and one-sided *t*-test were used in the CD level analysis and relative ion leakage analysis, respectively (^*^, *P* < 0.05; ^***^, *P* < 0.001). Error bars indicate the standard deviations.

Our previous study showed that StMPK7 promotes plant immunity through SA-related immune signalling [35]. We thus determine whether UBA2a/b-mediated plant immunity depends on the SA signalling pathway. The expression levels of two well-known SA-related genes, *pathogenesis-related protein-1 (PR1)* and *PR2*, were measured by qRT-PCR with the samples transient-expressing *StUBA2a/b-GFP* and *GUS-GFP* control. The results showed that both *NbPR1* and *NbPR2* are upregulated in *StUBA2a/b*-expressed samples (Fig. 3a). Consistently, the expressions of *NbPR1* and *NbPR2* were significantly decreased in *NbUBA2a/b*-silenced plants compared with the control TRV-*GUS* under both *P. infestans* infected (Fig. S6a, see online supplementary material) and uninfected (Fig. 3b) conditions. Moreover, silencing of *SlUBA2a/b* in tomato reduced the expression levels of *SlPR1* and *SlPR2* (Fig. S6b, see online supplementary material). These results indicate a positive regulator role of UBA2a/b in SA signalling pathway.

StUBA2a/b is reported to induce hypersensitive-like cell death in *N. tabacum* leaves [30]. As shown in Fig. 3c, our results showed that transient expression of StUBA2a/b (StUBA2a/b-GFP) also induced cell death at 7 days post-agro-infiltration (dpi) in *N. benthamiana*. Our previous study showed that CA-StMPK7 induced SGT1 (Suppressor of the G2 allele of Skp1)-dependent cell death [35], we thus examined whether the cell death induced by StUBA2a/b is dependent on SGT1. By transient expressing StUBA2a/b-GFP in TRV-*NbSGT1* and TRV-*GUS* plants, we observed that StUBA2a/b-induced cell death is abolished in *NbSGT1*-silenced plants (Fig. 3d and e), suggesting a common signalling component is depended by StUBA2a/b and CA-StMPK7.

To test whether StUBA2a/b-induced cell death requires SA-related signalling, as CA-StMPK7 does. We co-expressed salicylate hydroxylase NahG or control GFP with StUBA2a/b and measured the cell death by statistical analysis or ion leakage assay. Both results showed that the cell death is suppressed significantly by NahG (Fig. 3f–h). These results suggest that *UBA2a/b* triggered plant defence is involved in the SA-related signalling.

**Figure 4:**
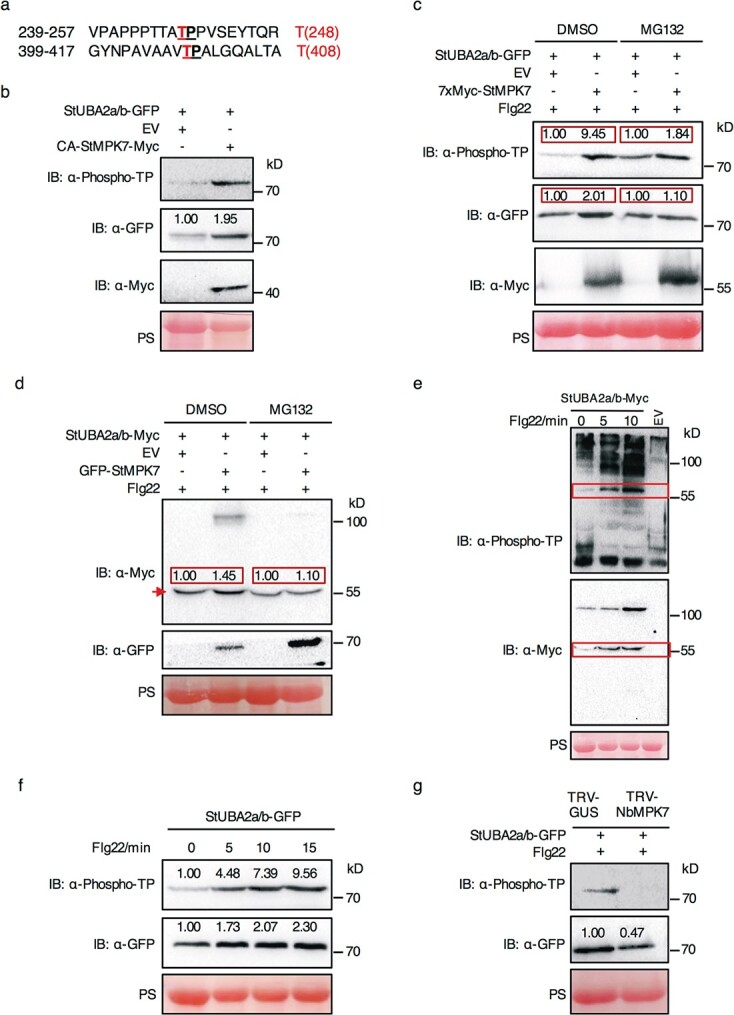
StUBA2a/b is phosphorylated and stabilized by StMPK7. **a** Amino acid sequence containing the two putative MAPK phosphorylation sites proline-directed threonine 248 and 408. **b** StUBA2a/b was phosphorylated and stabilized by CA-StMPK7 in vivo. Proteins were derived from *N*. *benthamiana* expressing StUBA2a/b with CA-StMPK7 or the EV control and extracted at 2 dpi. StUBA2a/b-GFP (**c**) and StUBA2a/b-Myc (**d**) were phosphorylated and stabilized by StMPK7 upon treatment with 10 μM flg22. 100 μM MG132 and the control DMSO (0.1%) were infiltrated into the leaves 12 hours before sample harvesting. Leaves were treated with 10 μM flg22 for 10 min and then subjected to protein extraction at 2 dpi. Red arrows indicate the intact protein of StUBA2a/b-Myc. StUBA2a/b-Myc (**e**) and StUBA2a/b-GFP (**f**) were increasingly phosphorylated and stabilized after flg22 treatment. At 2 dpi the infiltrated leaves were treated with flg22 and subsequently total proteins were extracted at 0, 5, 10, and 15 min after flg22 treatments, respectively. Empty-vector (EV) expressed in *N. benthamiana* serves as a control to rule out unspecific bands. The boxes with red lines mark the specific intact protein of StUBA2a/b-Myc. **g** Silencing of *NbMPK7* decreased the phosphorylation and stabilization of StUBA2a/b. StUBA2a/b was expressed in TRV-NbMPK7 or TRV-GUS leaves. The leaves were treated with flg22 for 10 min before total protein extraction. Anti-phospho-threonine-proline antibody (α-Phospho-TP) was used to detect the phosphorylation in proline-directed threonine sites. The presence and absence of proteins were indicated by + and -, respectively. Ponceau staining (PS) of the Rubisco was used to indicate the protein loadings. Numbers above the band signified the relative intensity of StUBA2a/b-GFP proteins normalized to Rubisco. In **b**, **c**, **f**, and **g**, only the bands of the intact proteins were shown and the full pictures of immunoblots were presented in Fig. S7, see online supplementary material.

**Figure 5:**
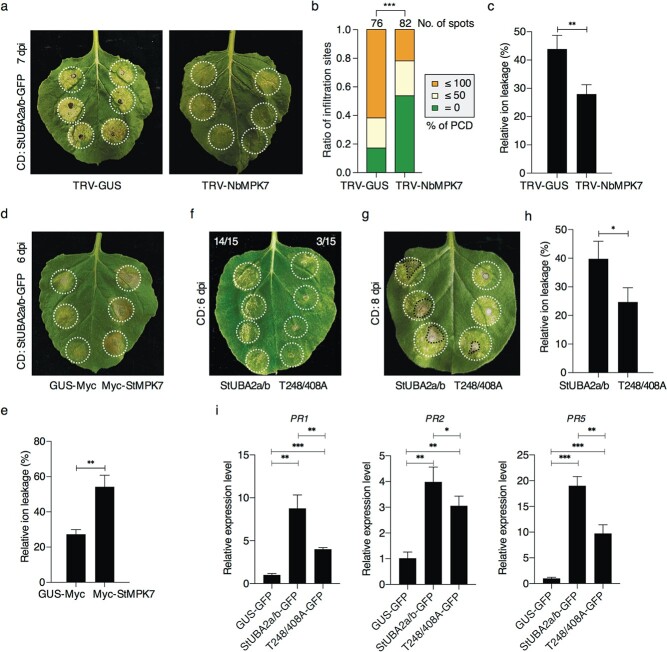
The phosphorylation of StUBA2a/b by MPK7 enhance the function of StUBA2a/b. Phenotype (**a**), statistical analysis of cell death (CD) levels (**b**) and relative ion leakage (**c**) of CD induced by StUBA2a/b-GFP in TRV-*NbMPK7* and the control TRV-*GUS*. Three weeks post-agro-inoculation with TRV-*NbMPK7* or TRV-*GUS*, StUBA2a/b-GFP was transient-expressed in the leaves of TRV plants at an OD_600_ of 1.0. The pictures and CD levels were recorded at 7 dpi. The total number of infiltration sites in (**b**) is shown above the columns. Relative ion leakage was measured subsequently. Phenotype (**d**) and relative ion leakage assay (**e**) indicated the enhancement of StUBA2a/b-induced CD by StMPK7 at 6 dpi. StUBA2a/b-GFP was co-expressed with Myc-StMPK7 or the control GUS-Myc on the right and left sides in more than 10 leaves of *N*. *benthamiana*. The phenotype of CD induced by StUBA2a/b-GFP or T248/408A-GFP in *N. benthamiana* leaves at 6 dpi (**f**) and 8 dpi (**g**). The ratios on the top of the leaves in (**f**) indicate the numbers of infiltration sites that CD has appeared versus the total number of infiltration sites at 6 dpi. **h** Barplot indicates the relative ion leakage of StUBA2a/b and T248/408A samples at 8 dpi. The white circles mark agro-infiltration area. The black dotted lines mark the area that has dried out. The CD level analysis is carried out by one-sided Wilcoxon rank-sum test (^***^, *P* < 0.001). In ion leakage analyses, one-sided *t*-tests are used (^*^, *P* < 0.05; ^**^, *P* < 0.01). (**i**) The relative expression levels of *PR* genes (*PR1*, *PR2*, and *PR5*) in samples expressing GUS-GFP, StUBA2a/b-GFP, or T248/408A-GFP. RNAs were extracted from *N. benthamiana* leaves expressing GUS-GFP, StUBA2a/b-GFP, or T248/408A-GFP for 2 days (each sample was prepared with four leaves). *NbACTIN* was used for normalization. Statistical analyses were performed using one-sided *t*-tests (^*^, *P* < 0.05; ^**^, *P* < 0.01; ^***^, *P* < 0.001). Error bars indicate the standard deviations.

### StUBA2a/b is phosphorylated and stabilized by StMPK7 to regulate the plant immunity

Threonine or Serine followed by Proline (TP or SP) served as the conserved consensus site for MAPK phosphorylation [37, 38]. To explore whether StUBA2a/b is the downstream substrate of StMPK7, we firstly examined the sequence of StUBA2a/b and found it has two potential proline-directed MAPK phosphorylation sites (T248, T408) (Fig. 4a).

Next, we detected whether StUBA2a/b is directly phosphorylated by CA-StMPK7 *in vivo* using an anti-phospho-threonine-proline (TP)-specific antibody. We coexpressed StUBA2a/b with CA-StMPK7 or empty vector (EV) in *N. benthamiana* and extracted the proteins at 2 dpi for subsequently immune blotting. As expected, TP phosphorylation of StUBA2a/b-GFP protein was found in CA-StMPK7-coexpressed samples but almost not in the control EV-coexpressed samples (Figure 4b). Besides, the accumulation of StUBA2a/b-GFP protein was significantly increased by co-expression with CA-StMPK7 (Fig. 4b, the full pictures in Fig. S7a, see online supplementary material; biological repeat in Fig. S7b, see online supplementary material). To confirm the phosphorylation and stabilization of StUBA2a/b by StMPK7, we coexpressed StUBA2a/b with StMPK7 or empty vector (EV) in *N. benthamiana* and treated the samples with flg22, which activates the phosphorylation of StMPK7 [35]. The following immune blotting showed that StMPK7 significantly enhanced the phosphorylation and protein accumulation of StUBA2a/b (Fig. S7c, see online supplementary material). To further investigate whether the stabilization of StUBA2a/b by StMPK7 is associated with 26S proteasome pathway, the 26S proteasome inhibitor MG132 or control DMSO was infiltrated into the leaves 12 hours before sample harvesting. The western blot analysis indicated that the phosphorylation of StUBA2a/b was enhanced by StMPK7 in both MG132 and DMSO samples (Fig. 4c and Fig. S7d, see online supplementary material). However, the stabilization of StUBA2a/b by StMPK7 is not observed in MG132-treated samples (Fig. 4c and d and Fig. S7d, see online supplementary material), suggesting the stabilization of StUBA2a/b by StMPK7 is directly or indirectly dependent on the 26S proteasome pathway. We further detect the phosphorylation and protein accumulation levels of StUBA2a/b during a treatment with flg22 for 0, 5, 10, and 15 min, respectively. As shown in Fig. 4e and f, both StUBA2a/b-Myc and StUBA2a/b-GFP were increasingly phosphorylated and stabilized after flg22 treatment. Empty vector (EV)-expressed sample was used as a control to rule out the nonspecific immunoblotting of the phospho-TP band in the total proteins (Fig. 4e). To detect whether the phosphorylation and stabilization levels were affected in *MPK7*-silenced plants (Fig. S3c, see online supplementary material), StUBA2a/b was expressed in TRV-*NbMPK7* or TRV-*GUS* plants and flg22 was infiltrated into the leaves for 10 min before sampling. As expected, both the phosphorylation and protein accumulation of StUBA2a/b was significantly decreased in TRV-*NbMPK7* plants (Fig. 4g and Fig. S7e, see online supplementary material). These results confirm that StUBA2a/b is phosphorylated and stabilized by StMPK7.

By phosphorylating and stabilizing StUBA2a/b, StMPK7 is supposed to activate or enhance the function of StUBA2a/b. To confirm this, we performed StUBA2a/b-induced cell death assays in *NbMPK7*-silenced plants. As expected, StUBA2a/b-induced cell death was significantly impaired in the *NbMPK7*-silenced *N. benthamiana* at 7 dpi (Fig. 5a–c). This result suggests that StUBA2a/b-induced cell death relies on MPK7. Consistently, when co-expressing with Myc-StMPK7 (Fig. S2c, see online supplementary material), the StUBA2a/b induced stronger cell death at 6 dpi (Fig. 5d and e) compared with that co-expressing with the control GUS-Myc. To investigate whether the phosphorylation of StUBA2a/b by StMPK7 is required for the function of StUBA2a/b, we constructed the substitution mutant StUBA2a/b^T248/408A^ (T248/408A for short) by replacing threonine 248 and 408 residues (the two putative phosphorylation sites) with alanine, which inactivates the phosphorylation site. Immunoblots showed that T248/408A could not be phosphorylated and hardly be stabilized by StMPK7 (Fig. S8, see online supplementary material). The subsequently transient expressions of StUBA2a/b-GFP and T248/408A-GFP (Fig. S2c, see online supplementary material) in *N. benthamiana* showed that, at 6 dpi, the cell death induced by StUBA2a/b-GFP appeared while T248/408A-GFP hardly induced any cell death (Fig. 5f). At 8 dpi, the cell death induced by StUBA2a/b was nearly dried out, in contrast, T248/408A-GFP induced only yellowish and weak cell death (Fig. 5g and h). These results suggest that the abolishment of phosphorylation sites delays and suppresses the cell death induced by StUBA2a/b. Besides, qRT-PCR results showed that T248/408A could still increase the *PR* gene expressions, however, with a significantly lower level as compared with StUBA2a/b (Fig. 5i). Taken together, we conclude that the function of StUBA2a/b is enhanced via phosphorylation by MPK7.

### StUBA2a/b is the downstream signalling component of StMPK7

**Figure 6:**
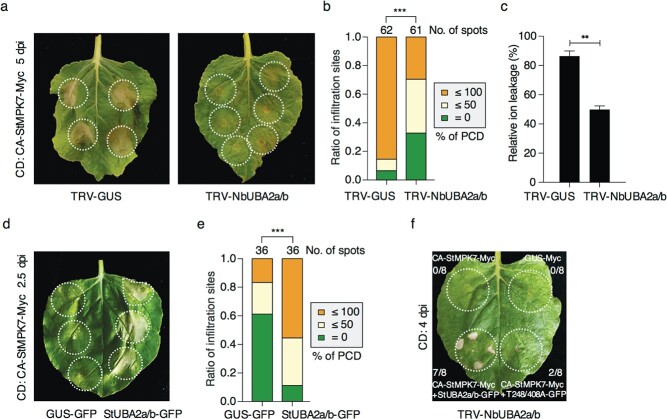
StUBA2a/b acts as the downstream signalling component of StMPK7. **a**–**c** The cell death induced by CA-StMPK7 is dependent on UBA2a/b. CA-StMPK7-induced CD was suppressed in *NbUBA2a/b*-silenced *N*. *benthamiana* as indicated by the phenotype (**a**), CD level analysis (**b**) and relative ion leakage assay (**c**). CA-StMPK7-Myc was infiltrated into the leaves of TRV-*NbUBA2a/b* or TRV-*GUS* plants at an OD_600_ of 0.7. CD was recorded and measured at 5 dpi. **d**, **e** CA-StMPK7-Myc was co-expressed with StUBA2a/b-GFP or the control GUS-GFP. The phenotype (**d**) and CD levels (**e**) show that StUBA2a/b increased the cell death triggered by CA-StMPK7. Photos were taken at 2.5 dpi. **f** The cell death induced by CA-StMPK7 was complemented by StUBA2a/b but not by T248/408A in TRV-*NbUBA2a* leaves at 4 dpi. CA-StMPK7-Myc (OD_600_ = 0.4) was co-expressed with StUBA2a/b-GFP or T248/408A-GFP in TRV-*NbUBA2a* leaves, respectively. CA-StMPK7-Myc or GUS-Myc was expressed as negative controls. The ratios on the leaves in (**f**) indicate the number of infiltration sites that CD has appeared versus the total number of infiltration sites. The total number of infiltration sites was shown above the columns in (**b**, **e**). The white circles mark agro-infiltration area. Statistical analyses were carried out by one-sided Wilcoxon rank-sum tests in (**b**, **e**) and one-sided *t*-test in (**c**) (^**^, *P* < 0.01; ^***^, *P* < 0.001).

To confirm the role of StUBA2a/b as the downstream signalling component of StMPK7, we transient expressed CA-StMPK7-Myc (Fig. S2c, see online supplementary material) in the TRV-*NbUBA2a/b* and TRV-*GUS* plants to examine whether *UBA2a/b* is required for CA-StMPK7-induced cell death. Both statistical analysis of the cell death grades and ion leakage analysis indicated that CA-StMPK7-induced cell death was significantly impaired in the TRV-*NbUBA2a/b* leaves (Fig. 6a–c). This result genetically supports that the function of MPK7 is dependent on its downstream substrate UBA2a/b. Besides, as compared to GUS-GFP, StUBA2a/b could promote the CA-StMPK7-induced cell death as revealed by the cell death grade analysis, suggesting an enhancement of CA-StMPK7 function by its downstream substrate StUBA2a/b (Fig. 6d and e). To further confirm this, we coexpressed CA-StMPK7 with StUBA2a/b or T248/408A in TRV-*NbUBA2a/b* leaves, respectively. At 4 dpi, StUBA2a/b complemented CA-StMPK7-induced cell death while the T248/408A mutant did not (Fig. 6f). These results suggest that the function of CA-StMPK7 is dependent on phosphorylating its substrate StUBA2a/b at T248/408 sites.

## Discussion

StMPK7 is the downstream target of StMKK1 and positively regulates plant defence via the SA signalling pathway [35]. What is the substrate of StMPK7 remains unknown. Here, we identified the RNA binding protein StUBA2a/b as the downstream substrate of StMPK7. LCI and co-IP assays showed the interaction between StUBA2a/b and StMPK7 or CA-StMPK7 (Fig. 1b–e and Fig. S2, see online supplementary material). Immune blotting with phospho-threonine-proline antibody indicates StUBA2a/b is phosphorylated by CA-StMPK7, and this phosphorylation stabilized StUBA2a/b *in planta* (Fig. 4b and Fig. S7a and b, see online supplementary material). Our previous study has shown that flg22 induces the phosphorylation of StMPK7 [35]. As expected, StUBA2a/b is also phosphorylated by StMPK7 upon flg22 treatment (Fig. 4c and d and Fig. S7c and d, see online supplementary material). Besides, the phosphorylation and protein accumulation levels of StUBA2a/b are increasingly enhanced after flg22 treatment (Fig. 4e and f), suggesting the phosphorylation and stabilization of StUBA2a/b are correlated with the activation of MPK7. Consistently, silencing of *NbMPK7* nearly abolished the phosphorylation and significantly repressed the stabilization of StUBA2a/b (Fig. 4g), further confirming the requirement of MPK7 in phosphorylation and stabilization of StUBA2a/b. Moreover, the cell death induced by StUBA2a/b was impaired by silencing of *NbMPK7*, but was enhanced by co-expressing with StMPK7 (Fig. 5a–e), suggesting the phosphorylation of StUBA2a/b by StMPK7 may activate or enhance its function. The phosphorylation sites disabled mutant T248/408A induced a delayed and weaker cell death than StUBA2a/b (Fig. 5f–h). Consistently, the elevation of *PR* gene expressions induced by T248/408A was also significantly decreased as compared with StUBA2a/b (Fig. 5i). These results further indicate that phosphorylation by StMPK7 enhances the function of StUBA2a/b. Taken together, it is clear that StUBA2a/b is the downstream substrate of StMPK7 and the phosphorylation by StMPK7 enhances its functions. MAPKs have been reported to phosphorylate different substrates, most of which are transcription factors, whereby to regulate diverse biological processes [8]. Notably, increasing evidence showed that some RNA binding proteins (RBPs) are also the substrates of MAPK cascades [24, 39]. Hence, MAPKs can participate in mRNA regulation not only by targeting transcription factors, but also by targeting RBPs. For example, yeast RBP Rnc1 is phosphorylated by Pmk1 [37]. In human, hnRNP-K is a substrate of MEK1 and its phosphorylation is required for regulating mRNAs translation [40]. Tristetraprolin (TTP), one of the best studied RBPs in mammals, is phosphorylated by p38 MAPK [41]. In plants, studies about RBPs as the substrates of MAPKs are rare. To our knowledge, only one RBP in Arabidopsis, tandem zinc finger protein 9 (TZF9), is shown phosphorylated by MPK3/6 [23, 24]. Our study revealed a distinct type of RBP, UBA2a/b, is a novel type of substrate phosphorylated by StMPK7.

Our study shows that StUBA2a/b is stabilized by StMPK7 (Fig. 4) while the stabilization of StUBA2a/b^T248/408A^ by StMPK7 is largely reduced (Fig. S8, see online supplementary material), suggesting the phosphorylation of StUBA2a/b at the phosphorylation sites T248/408 leads to the stabilization. This is consistent with many of the reports on the protein stability alteration affected by MAPKs phosphorylation [8]. For example, the phosphorylation of AHL13 (AT-hook motif containing nucleus localized DNA-binding protein) by MPK6 leads to the stabilization and thereby regulates the immune function of AHL13 [42]. Several substrates of MPK3/6, including ACS2/6 [12], ERF6 [14] and SPL [43], are all stabilized via the phosphorylation by MPK3/6. In contrast, some other MAPK substrates were shown destabilized via phosphorylation, such as WRKY46 [44], the substrate of MPK3, and TZF9, the substrates of MPK3/6 [23, 24]. In our study, stabilization of StUBA2a/b by StMPK7 is not observed anymore in MG132-treated samples (Fig. 4c and d), suggesting an association with the 26S proteasome pathway. It’s noted that StUBA2a/b protein accumulation was decreased but not increased upon MG132 treatment, suggesting StUBA2a/b may also be involved in other degradation pathway, for example, the autophagy pathway, which is reported to be activated by MG132 treatment [45]. We thus supposed that the stabilization of StUBA2a/b by StMPK7 may directly or indirectly depend on the 26S proteasome pathway.

AtUBA2a, AtUBA2b, and StUBA2a/b are able to regulate the expression of some wounding and senescence-associated genes, as well as some defence-related genes [29, 30], but the role of UBA2a/b in plant defence has not been defined. In this study, inoculation tests showed that overexpression of *StUBA2a/b* enhanced plant resistance to *Phytophthora* pathogens while silencing of *NbUBA2a/b*, *SlUBA2a/b*, or knockout of *AtUBA2a* decrease the resistance, suggesting a conserved positive regulator role of UBA2a/b in plant defence (Fig. 2). A number of RBPs have been shown involved in regulating plant defence. For example, AtRBP-DR1 is involved in the SA signalling pathway and thus regulates plant immunity [46]. AtGRP7, one RBP identified as the host target of a bacterial pathogen effector, is required for plant defence [47, 48]. A nucleo-cytoplasmic RBP LIF2 is involved in suppressing the plant immune response [49]. Our study further uncovered the role of RBP UBA2a/b in regulating plant immunity.

In our previous study, we have revealed that StMPK7 regulates plant defence via the SA signalling pathway [35]. As the substrate of StMPK7, StUBA2a/b was supposed to be involved in the same pathway. As expected, qRT-PCR data indicated that StUBA2a/b positively regulates the expressions of SA-related marker genes *PR1* and *PR2* (Fig. 3a and b). Besides, the cell death induced by StUBA2a/b is also dependent on SGT1 and suppressed by NahG (Fig. 3c–h), similar to CA-StMPK7 [35]. It is reported that overexpression of *AtUBA2a*/*AtUBA2b* induced hypersensitive-like cell death and leaf yellowing via enhancing the senescence and defence response pathways [29]. Besides, StUBA2a/b is shown to induce the same phenotype in Arabidopsis and *N. tabacum* leaves as AtUBA2a/AtUBA2b do [30]. Moreover, the StUBA2a/b transgenic Arabidopsis plants could increase the H_2_O_2_ accumulation, SA content and SA-related gene expressions [30]. Our results are consistent with these studies and further clarify the role of UBA2a/b in plant immunity. The attenuation of CA-StMPK7-induced cell death in *NbUBA2a/b*-silenced plants genetically indicates the role of UBA2a/b as the downstream component of StMPK7 (Fig. 6a–c). We thus underline that StMPK7 phosphorylates and stabilizes its downstream component StUBA2a/b, which is involved in the SA signalling pathway, to regulate plant defence.

## Materials and methods

### Plasmid construction

Full-length StUBA2a/b was amplified by PCR with the cDNA of potato cultivar Desiree as a template and cloned into pART27-GFP, pART27-Myc, and pCAMBIA-Cluc, respectively. StMPK7 was amplified and inserted into pCAMBIA-Nluc. The 300 bp cDNA fragment of *NbUBA2a/b* or *SlUBA2a/b* was amplified and inserted into tobacco rattle virus TRV2 vector. StUBA2a/b^T248/408A^ was amplified by site-directed mutagenesis PCR. All primers are listed in Table S2, see online supplementary material.

### Microbe and plant cultivation

*Agrobacterium tumefaciens* strain C58C1 and *Escherichia coli* DH5a were routinely cultured in Luria Bertani (LB) media at 28°C and 37°C, respectively. *P. infestans* strain 14-3-GFP [50] was grown on rye sucrose agar at 18°C and *P. capsici* strain LT263 was grown on carrot agar medium at 23°C. Transgenic potato StMPK7 OE lines and *N. benthamiana* plants were grown as described in our previous study [35].

### Co-IP and LC–MS/MS analysis

The proteins were extracted and incubated with GFP-trap_A beads (Chromotek, Planegg-Martinsried, Germany) for 3 hours at 4°C, followed by the removal of the supernatant and washing with dilution buffer consisting of 10 mM Tris–HCl (pH 7.5), 150 mM NaCl, and 0.5 mM EDTA. The binding proteins were eluted and boiled in sample buffer for 10 min at 95°C. The proteins enriched by immunoprecipitation are concentrated and desalted with ultrafiltration membrane. After that, the proteins were digested with trypsin and subjected to analysis by high-sensitivity LC–MS/MS (QExactive HF-X, ThermoFisher, Waltham, MA, USA).

### Western blot assays

Total proteins were extracted with lysis buffer as described previously [33] and subsequently fractionated through sodium dodecyl sulfate-polyacrylamide gel electrophoresis (SDS-PAGE). Immunodetections with the corresponding antibodies were performed to detect the proteins that transferred onto the polyvinylidene difluoride (PVDF) membranes by electroblotting.

### Phosphorylation assay

Threonine phosphorylation of StUBA2a/b was detected by western blotting using the antibody phospho-threonine-proline mouse mAb (P-Thr-Pro-101, #9391, Cell Signalling Technology, Massachusetts, USA). The proteins used for phosphorylation assay were extracted with the lysis buffer containing phosphatase inhibitor cocktails 2 and 3 (P5726 and P0044, Sigma, USA).

### Firefly LCI assay

Agrobacterium containing StMPK7-Nluc and Cluc-StUBA2a/b or CA-StMPK7-Nluc and Cluc-StUBA2a/b was co-infiltrated into *N. benthamiana* leaves and the LUC images were captured at 2 days post-agro-infiltration as previously described [51]. Co-expression of StMPK7-Nluc with Cluc, CA-StMPK7-Nluc with Cluc or Nluc with Cluc-StUBA2a/b was used as the negative controls.

### Virus-induced gene silencing

The *NbMPK7*-silenced plants were constructed as described previously [35]. For the construction of *NbUBA2a/b*-silenced plants, a 300 bp fragment of Niben101Scf05519g01004.1 and Niben101Scf01001g07022.1, the two copies of *StUBA2a/b*, was designed with the VIGS tool [52] and inserted into the TRV2 vector. TRV2 vector containing a *GUS* fragment was used as a control. Agrobacterium cultures containing TRV1 and TRV2 were infiltrated into the first two leaves of six-leaf stage *N. benthamiana* with a final concentration of OD_600_ = 1. The silencing effect was monitored through *PDS*-silenced plants. The *SlUBA2a/b*-silenced tomato plants were prepared as the similar assays in *N. benthamiana*. A 300 bp fragment of Solyc01g008970.4.1 designed by the VIGS tool was inserted into the TRV2 vector for silencing.

### Agrobacterium infiltration

The *A. tumefaciens* strain C58C1 carrying different constructs was cultured in liquid LB medium with appropriate antibiotics for 24 hours. The agrobacterium cells were centrifuged and then resuspended in infiltration buffer (10 mM MgCl_2_, 200 μM acetosyringone, 1 mM MES, pH 5.6) to the appropriate ratio.

### Plant cell death assay

StUBA2a/b-GFP, T248/408A-GFP or CA-StMPK7-Myc was agro-infiltrated into *N. benthamiana* leaves to measure the cell death level. Both cell death grades [53] and ion leakage [50] were assayed as previously described. The cell death grades were divided into three levels according to the percentage of cell death area (0%, 0–50%, and 50–100% cell death).

### Pathogen infection assays in *N. benthamiana*, Arabidopsis, tomato, and potato

The detached leaves of *N. benthamiana* were used for inoculation. About 1500 zoospores from *P. infestans* strain 14–3-GFP or 3 mm^2^ mycelium plugs cut from the *P. capsici* strain LT263 cultures were inoculated onto the *N. benthamiana* leaves. Lesions developed in the leaves were measured at 6–7 days after *P. infestans* infection and 2–3 days after *P. capsici* infection. Four-week-old Arabidopsis leaves were infected with *P. capsici* zoospore suspension (400–500 zoospores per infection site). The leaves of tomato were inoculated with *P. capsici* or *P. infestans* zoospore suspension (600–800 zoospores per infection site) at 4 weeks post agro-inoculation with TRV vectors. Potato plants cultured in MS medium for 2 weeks were transformed into *R. solanacearum* suspensions as described previously [54]. Wilting symptoms were photographed at 6 dai and the bacterial growth was quantified by the bacterial colonies derived from the unit weight of potato plant in aerial parts.

### Real-time quantitative reverse transcription-PCR analysis

Plant samples were ground in liquid nitrogen and then subjected to RNA extraction using the RNA Kit (TianGen, Beijing, China, Cat No. DP419). cDNA was synthesized using a Real-Time Kit for qPCR (Accurate Biology, Hunan, China, Cat No. AG11705). *NbACTIN*, *AtACTIN* and *SlACTIN* were used as the reference genes in *N. benthamiana*, Arabidopsis and tomato, respectively, for normalization.

### Confocal microscopy imaging assay

To visualize the co-localization, *A. tumefaciens* carrying StUBA2a-GFP was co-infiltrated with StMPK7-mCherry or CA- StMPK7-mCherry into *N. benthamiana* leaves at a low concentration (OD_600_ = 0.1–0.2). The fluorescence was observed at 2 dpi using an Olympus FV3000 confocal microscope. GFP and mCherry were excited at 488 and 559 nm, respectively. The emissions of GFP and mCherry were detected at 500–530 and 580–630 nm, respectively.

### Accession numbers

StUBA2a/b: Soltu.DM.01G005860.9; StMPK7: Soltu.DM.08G028240.1; NbUBA2a/b.1: Niben101Scf01001g07022.1; NbUBA2a/b.2: Niben101Scf05519g01004.1; NbMPK7a: Niben101Scf00254g02007.1; NbMPK7b: Niben101Scf00421g01029.1; AtUBA2a: AT3G56860; SlUBA2a/b: Solyc01g008970.4.1; StMPK13: Soltu.DM.11G025980.1.

## Acknowledgements

We thank the Horticulture Science Research Center (Northwest A&F University) for experimental assistance. We thank Dr Hua Zhao and Dr Fengping Yuan (State Key Laboratory of Crop Stress Biology for Arid Areas, Northwest A&F University, Yangling, China) for confocal experimental assistance and are grateful to Dr Qiong Zhang and Technician Xiaona Zhou of CSBAA for assistance with high-sensitivity LC–MS/MS. This work was financed by the National Natural Science Foundation of China (32072401, 32102176), the Chinese Universities Scientific Fund (2452020145, 2452018028 and 2452017069), the Basic Research Project of Natural Science in Shaanxi Province (2021JQ-164), and the Programme of Introducing Talents of Innovative Discipline to Universities (Project 111) from the State Administration of Foreign Experts Affairs (B18042).

## Author contributions

Y.D. and T.L. designed the research. T.L., H.Z., L.X., X.C., J.F., and W.W. performed the experiments. T.L., Y.D., and H.Z. analysed the data. T.L. and Y.D. wrote the paper. All authors reviewed the paper.

## Data availability statement

All data generated in this study are presented in the paper and/or the Supplementary Data. Additional data related to this paper may be requested from the authors.

## Conflict of interest

The authors declare no conflicts of interest.

## Supplementary data

Supplementary data is available at *Horticulture Research * online.

## Supplementary Material

Web_Material_uhac177
