# Supplementary material for: StMPK7 phosphorylates and stabilizes a potato RNA-binding protein StUBA2a/b to enhance plant defence responses
Source: Hortic Res. 2022 Aug 24;9:uhac177. doi: 10.1093/hr/uhac177 (PMC9614683; doi:10.1093/hr/uhac177)
Supplement: Web_Material_uhac177 [file web_material_uhac177.zip › Li et al. Supplemental Information_clean.docx]

**Supplemental Information**


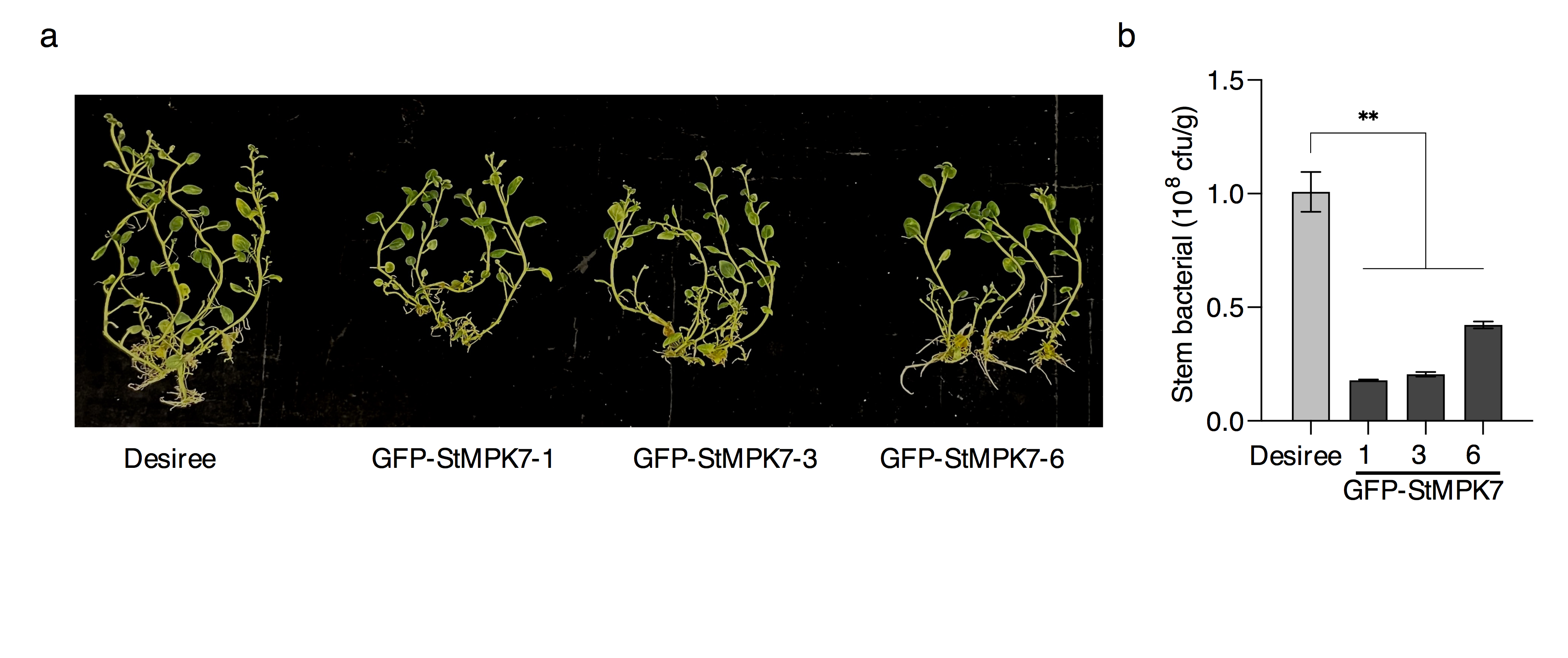


**Supplemental Figure 1. Overexpressing of *StMPK7* enhances potato defense against *Ralstonia solanacearum*.** (a) Representative images of the wilting symptoms developed in the wild-type Desiree and three *GFP-StMPK7* transgenic potato lines. (b) The barplot shows the number of bacteria in the potato stems. Desiree and transgenic potato lines were grown in MS medium for 2 weeks and then transferred into *R. solanacearum* suspension (1×10^8^ cfu/mL). The wilting symptoms were photographed at 6 dai and the number of bacteria was quantified subsequently. Four independent plants were used in the bacterial quantification. Error bars show the standard deviations. The significant difference is assessed by two-sided *t*-test (**, *P* < 0.01).


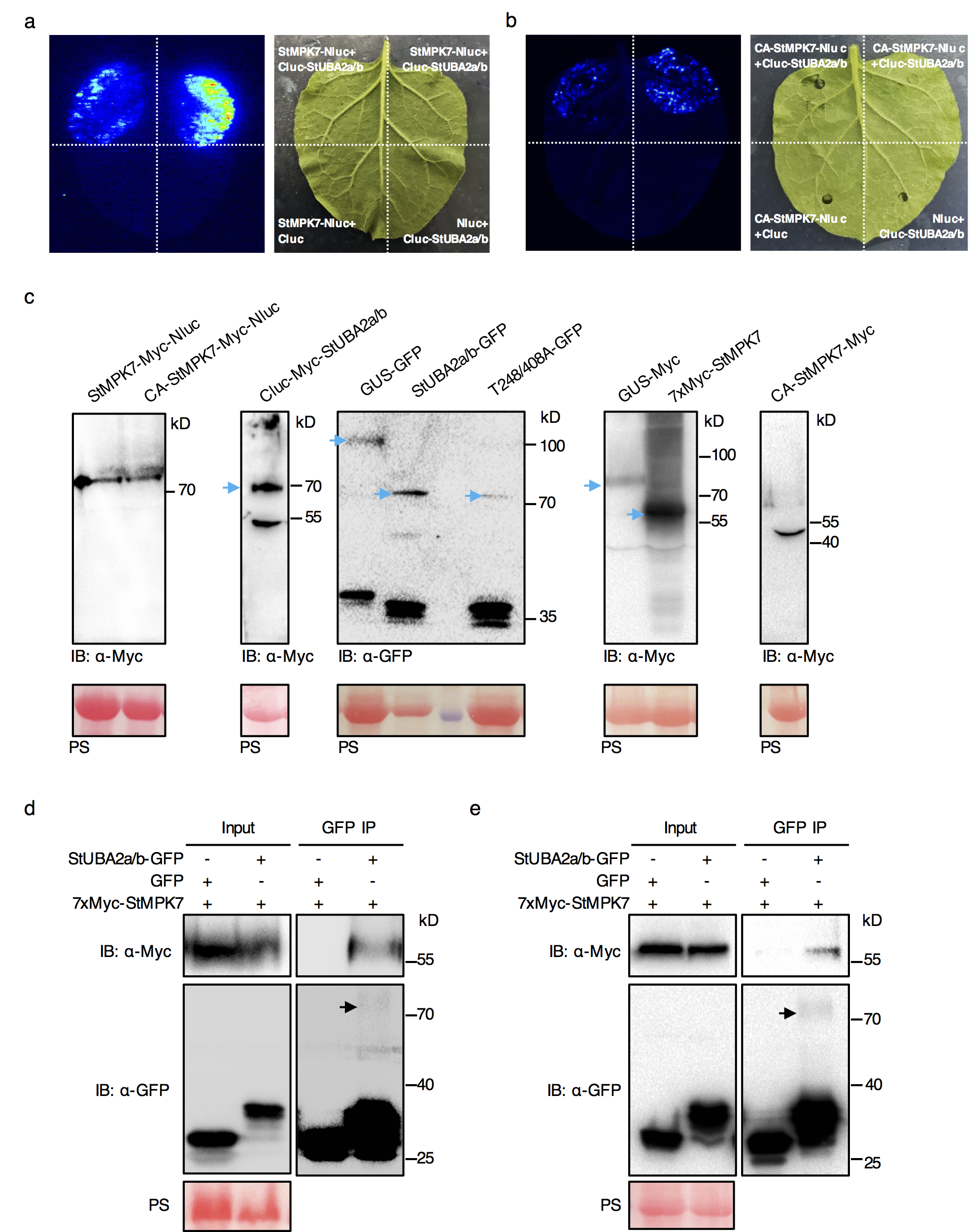


**Supplemental Figure 2. The interaction between** **StMPK7/CA-StMPK7 with** **StUBA2a/b and the protein expression examination.** StUBA2a/b interacts with both StMPK7 (a) and CA-StMPK7 (b) in LCI (the additional replicate as in the Figure 1b, c). Cluc-StUBA2a/b was coexpressed with StMPK7-Nluc or CA-StMPK7-Nluc in *N. benthamiana leaves*, respectively. The imagines were taken by CCD-camera (left panel) or by normal camera (right panel) at 2 dpi. (c) Immunoblots showing the protein expressions in transient expression assays. Total proteins were extracted from the *N. benthamiana* samples expressing the corresponding constructs as indicated at the top of the pictures at 2 dpi. The blue arrows mark the corresponding intact protein. (d-e) StUBA2a/b-GFP interacts with 7xMyc-StMPK7 in co-IP. (d) and (e) are two biological replicates of the co-IP assay. IP was performed via GFP-Trap beads. Black arrows indicate the intact protein of StUBA2a/b-GFP. Free GFP was used as the negative control. “+” and “-” indicates the presence and absence of the construct in the samples. The protein loading is shown by Ponceau staining (PS).

**Supplemental Figure 3.** **The** **silencing efficiency and plant** **morphology of** **TRV inoculated *N. benthamiana*.** (a) The expression level of *NbUBA2a/b* is significantly decreased in TRV-*NbUBA2a/b* plants as compared with the control TRV-*GUS* plants. RNA was extracted from the middle leaves of *N. benthamiana* inoculated with TRV-*NbUBA2a/b* or TRV-*GUS*. qRT-PCR was performed to measure the expression level of *NbUBA2a/b*. *NbACTIN* was used as the reference gene. Statistical analysis was performed with one-sided *t*-test (***, *P* < 0.001). Error bars represent the standard deviations. (b) The *NbUBA2a/b*-silenced *N. benthamiana* does not show any difference in morphology compared with the TRV-*GUS* control. The photographs were taken 3 weeks after agro-inoculation with TRV vectors. TRV-*PDS* (phytoene desaturase) was used to monitor the silencing process. (c) The expression level of *NbMPK7* in TRV-*NbMPK7* plants. TRV-GUS was used as the control.

**Supplemental Figure 4. Arabidopsis T-DNA insertion mutant *Atuba2a* shows increased plant susceptibility to *P. capsici*.** (a) Barplot shows the relative expression of *AtUBA2a* in the Arabidopsis T-DNA mutant. Total RNAs were extracted with the leaves of 4-week-old Col-0 and *Atuba2a* mutant. “nd” means no amplification detected. Error bars indicated the standard deviations. (b) The plant morphology of 5-week-old Col-0 and *Atuba2a*. (c) Representative leaves of Col-0 and *Atuba2a* infected by *P. capsici*. The pictures were photographed under normal light (upper panel) and blue light (lower panel) at 2 dai. (d) Barplot shows the analysis of lesion diameters developed in Col-0 and *Atuba2a* at 2 dai. Detached leaves from 7 independent Col-0 or *Atuba2a* plants were inoculated by *P. capsici* zoospore suspensions (300-500 zoospores per infection site). Four-week-old Arabidopsis plants were used in the inoculation assay. Statistical analysis was performed with one-sided *t*-tests (*, *P* < 0.05, n=7). The inoculation assays with *P. capsici* were repeated two times with similar results.


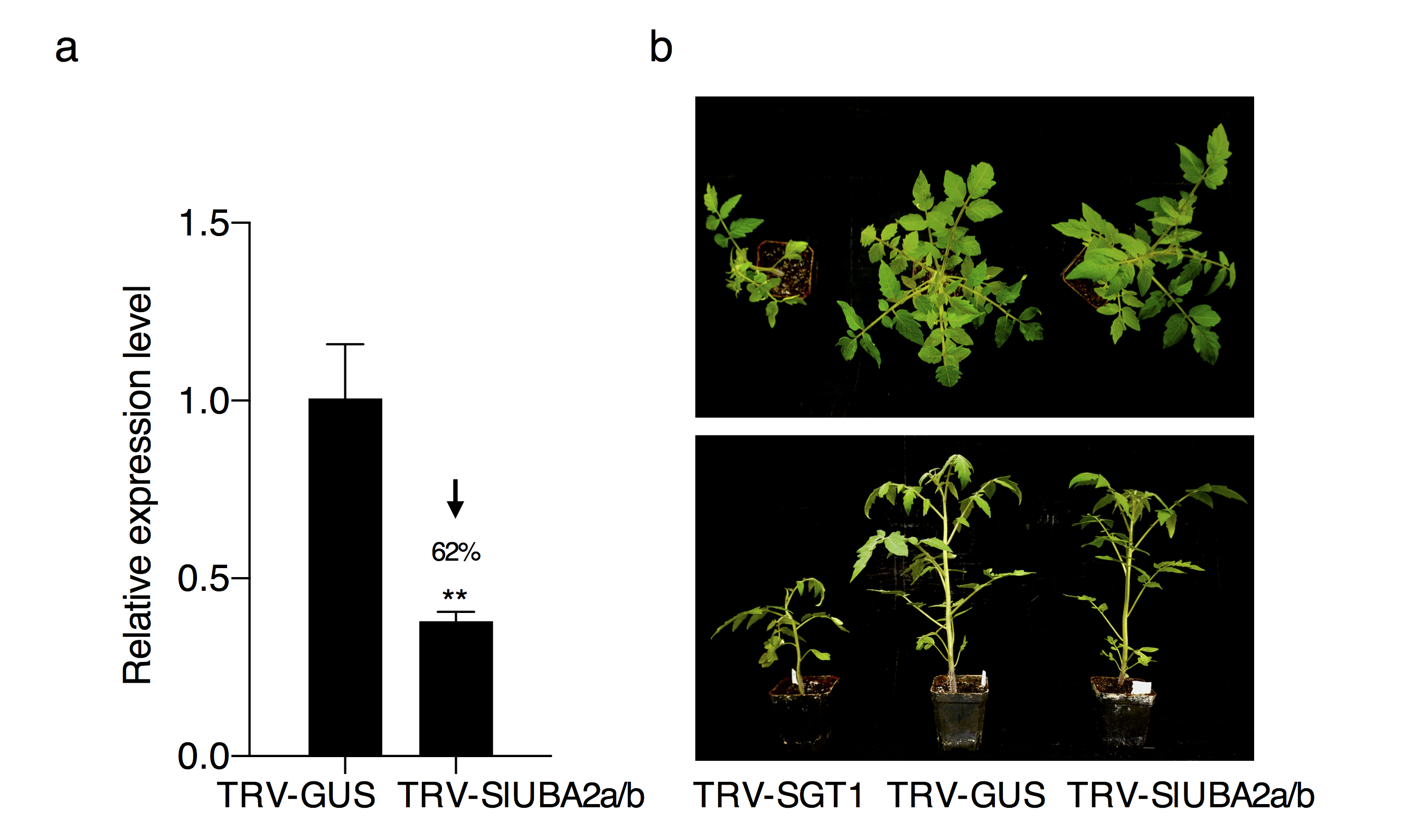


**Supplemental Figure 5. The silencing efficiency and plant morphology of *SlUBA2a/b*-silenced tomato.** (a) The relative expression level of *SlUBA2a/b* in TRV-*SlUBA2a/b* tomato plants. Total RNAs were derived from leaves of TRV-*SlUBA2a/b* or TRV-*GUS* plants 4 weeks after ago-infiltration with TRV vectors. Statistical analysis was performed with one-sided *t*-test (*, *P* < 0.05). Error bars represent the standard deviations. (b) Photos of the representative TRV-*SlUBA2a/b* and TRV-*GUS* plants. TRV-*SGT1* (phenotype dwarf) was used to monitor the silencing process. Photos were taken at 4 weeks after ago-infiltration with TRV vectors.


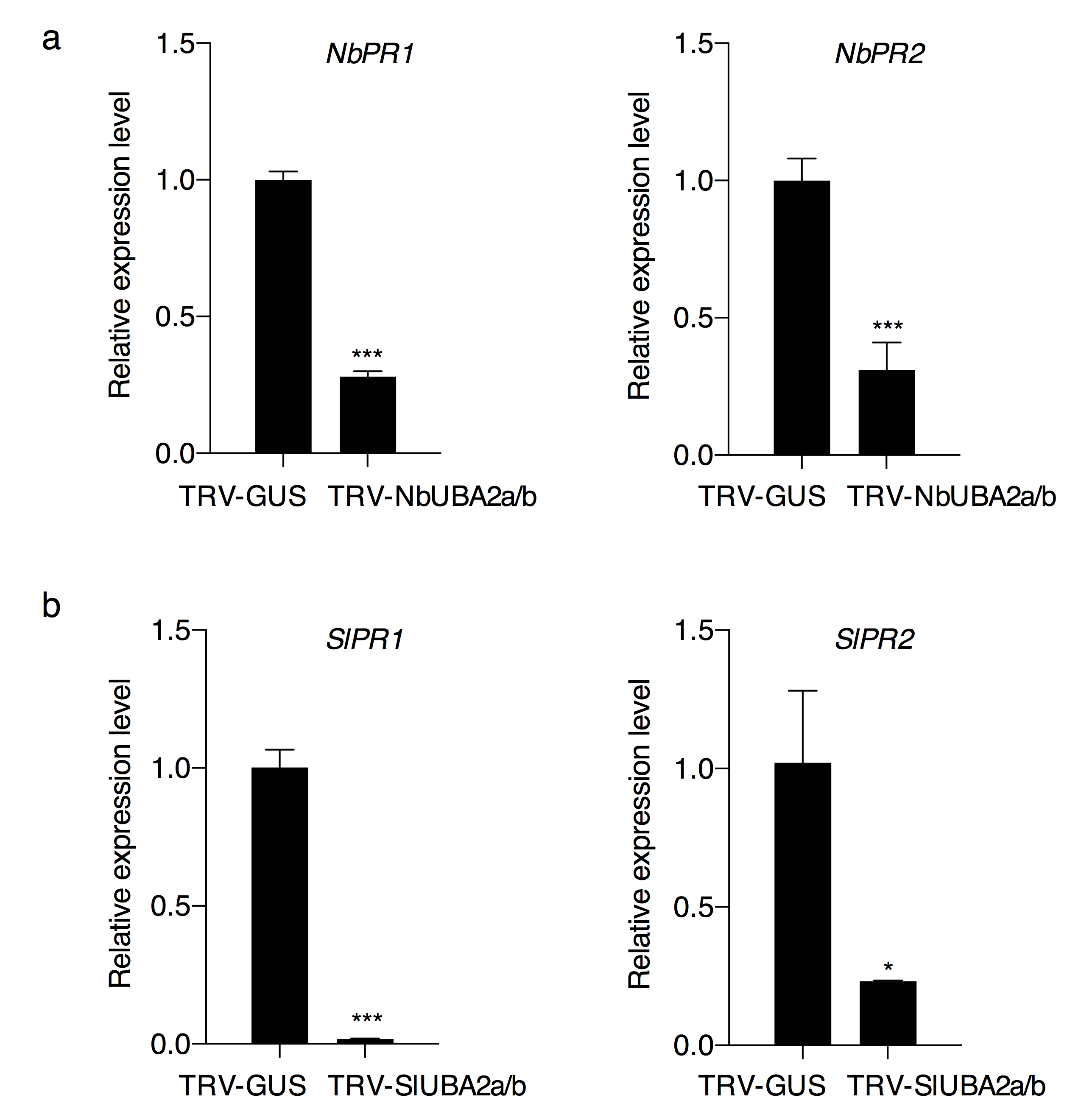


**Supplemental Figure 6. The relative expression levels of** ***PR* genes in** **TRV-*NbUBA2a/b* or TRV-*SlUBA2a/b* plants.** (a) Barplots show the relative expression levels of *NbPR1* and *NbPR2* in *NbUBA2a/b*-silenced leaves upon *P. infestans* infection. Total RNAs were derived from the TRV-*NbUBA2a/b* or TRV-*GUS* leaves inoculated with *P. infestans* for 6 days. *NbACTIN* was used as the reference gene for normalization. (b) The relative expression levels of *SlPR1* and *SlPR2* in *SlUBA2a/b*-silenced leaves. qRT-PCRs were performed with the samples of the silencing efficiency examination in Supplemental Figure 5a. *SlACTIN* was used for normalization. Statistical analysis was performed with one-sided t-tests (*, *P* < 0.05; ***, *P* < 0.001). Error bars represent the standard deviations.


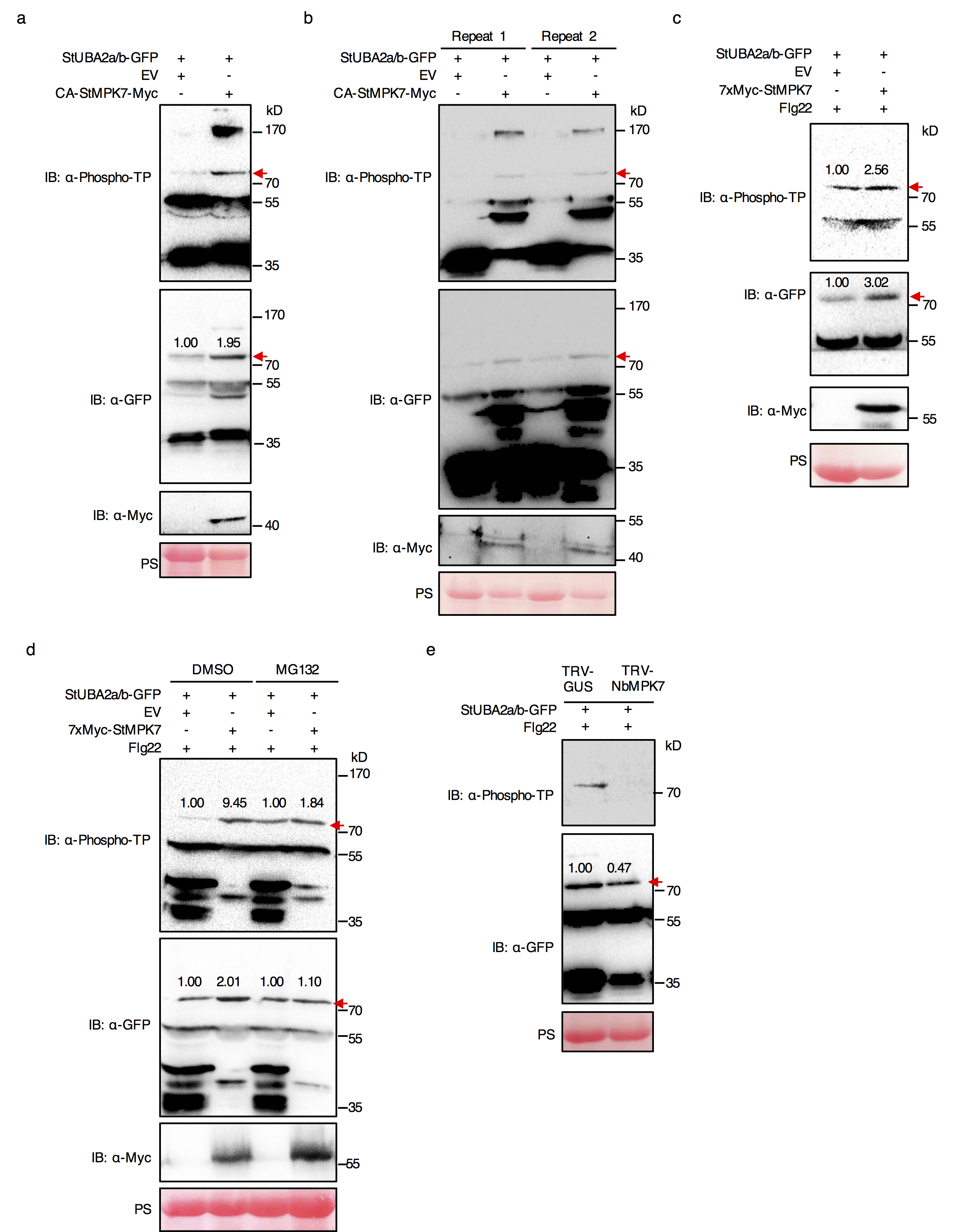


**Supplemental Figure 7.** **StUBA2a/b was phosphorylated and stabilized by CA-StMPK7 and StMPK7.** (a) Full pictures of Figure 4b. (b) The additional biological replicate (as in Figure 4b) showed the phosphorylation and stabilization of StUBA2a/b-GFP by CA-StMPK7-Myc. Total proteins were extracted with *N. benthamiana* leaves expressing StUBA2a/b-GFP with CA-StMPK7-Myc or the EV control at 2 dpi. Repeat 1 and repeat 2 indicate two technical replicates. (c) StUBA2a/b was phosphorylated and stabilized by StMPK7 (upon treatment with flg22). StUBA2a/b-GFP was coexpressed with 7xMyc-StMPK7 or EV control in *N. benthamiana* leaves. The samples were treated with 10 μM flg22 for 10 min and then subjected to protein extraction at 2 dpi. Full pictures of Figure 4c (d) and Figure 4g (e). Anti-phospho-threonine-proline antibody (α-Phospho-TP) was used to detect the phosphorylation in proline-directed threonine sites. The presence and absence of constructs in the samples were indicated by + and -, respectively. The protein loadings were indicated by Ponceau staining (PS). Numbers above the band show the relative intensity of StUBA2a/b-GFP proteins normalized to Rubisco. The red arrows mark the intact protein of StUBA2a/b-GFP.


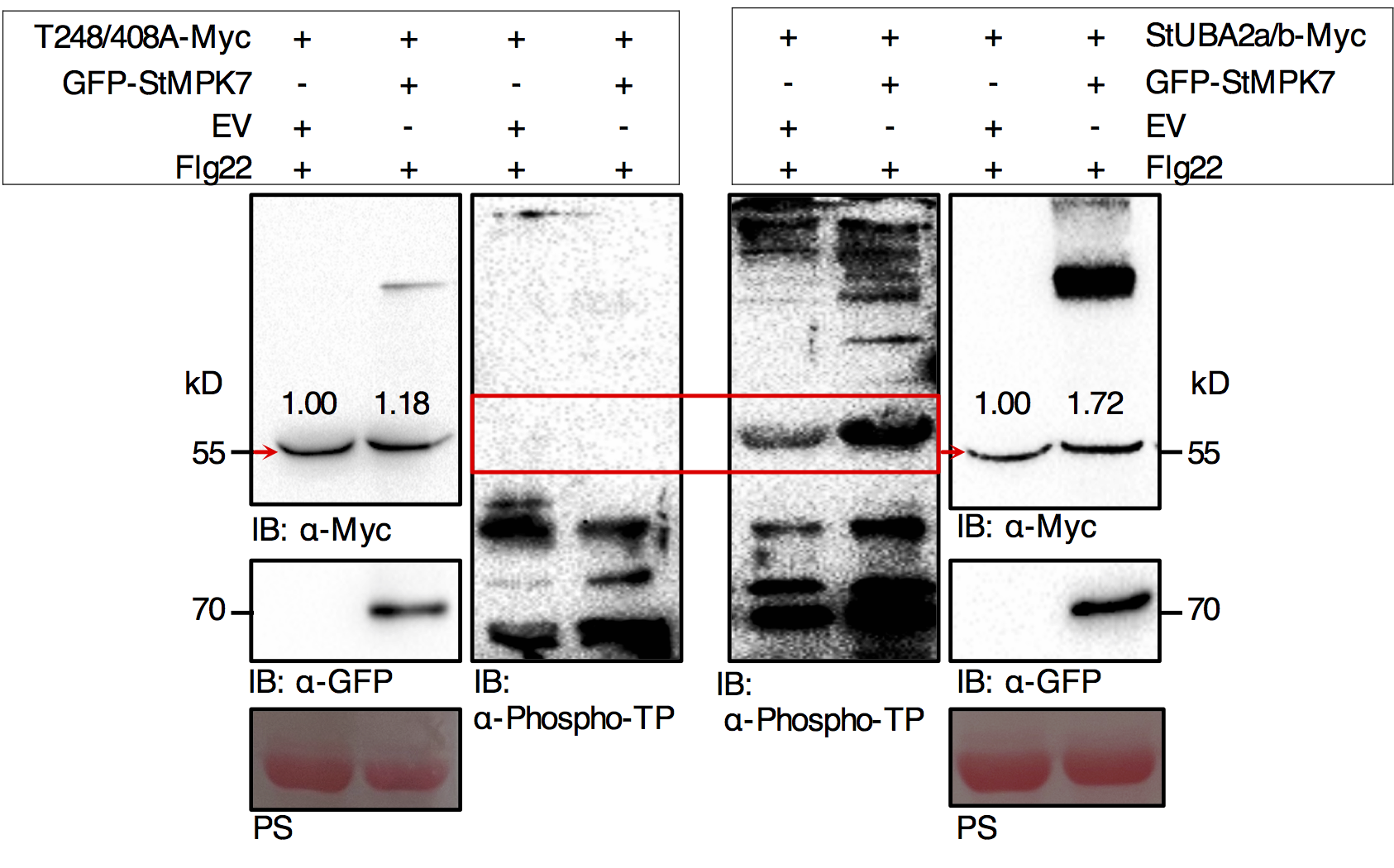


**Supplemental Figure 8. StUBA2a/b^T248/408A^ cannot be phosphorylated by StMPK7.** Total proteins were extracted with *N. benthamiana* leaves expressing StUBA2a/b^T248/408A^-Myc with GFP-StMPK7 or the EV control at 2 dpi. Co-expression of StUBA2a/b-Myc with GFP-StMPK7 was used as the positive control. Leaves were treated with 10 μM flg22 for 10 min before sampling. Red arrows indicate the StUBA2a/b^T248/408A^-Myc or StUBA2a/b-Myc protein. The box with red line marks the intact protein of StUBA2a/b-Myc in the anti-phospho-TP immunoblot. Numbers above the band signified the relative intensity of StUBA2a/b^T248/408A^-Myc or StUBA2a/b-Myc protein normalized to Rubisco. Ponceau staining (PS) of the Rubisco was used to indicate the protein loadings.

**Supplemental Table 1.** **Mass spectrometry datasheet of the potential StMPK7-interacting proteins.**

**Supplemental Table 2. Primers used in this study.**
